# Impact of age, leukocyte count and day 21-bone marrow response to chemotherapy on the long-term outcome of children with philadelphia chromosome-positive acute lymphoblastic leukemia in the pre-imatinib era: results of the FRALLE 93 study

**DOI:** 10.1186/1471-2407-9-14

**Published:** 2009-01-13

**Authors:** Virginie Gandemer, Marie-Francoise Auclerc, Yves Perel, Jean-Pierre Vannier, Edouard Le Gall, Francois Demeocq, Claudine Schmitt, Christophe Piguet, Jean-Louis Stephan, Odile Lejars, Marianne Debre, Philippe Jonveaux, Jean-Michel Cayuela, Sylvie Chevret, Guy Leverger, Andre Baruchel, the FRALLE group

**Affiliations:** 1 Department of Pediatric Hematology, University Hospital of Rennes, Rennes, France; 2 Department of Pediatric Hematology, University Hospital of St Louis, Paris, France; 3 Department of Pediatric Hematology, University Hospital of Bordeaux, Bordeaux, France; 4 Department of Pediatric Hematology, University Hospital of Rouen, Rouen, France; 5 Department of Pediatric Hematology, University Hospital of Clermont-Ferrand, Clermont-Ferrand, France; 6 Department of Pediatric Hematology, Nancy University Hospital, Vandoeuvre-les-Nancy, France; 7 Department of Pediatric Hematology, University Hospital of Limoges, Limoges, France; 8 Department of Pediatric Hematology, University Hospital of St Etienne, St Etienne, France; 9 Department of Pediatric Hematology, University Hospital of Tours, Tours, France; 10 Department of Pediatric Immunology and Hematology of Necker, Paris, France; 11 Department of Cytogenetics of University Hospital of Nancy, Nancy, France; 12 Laboratory of Hematology of University Hospital of St Louis, Paris, France; 13 Department of Statistics of St Louis/Université, Paris 7, France; 14 Department of Pediatric Hematology, University Hospital of Trousseau, Paris, France

## Abstract

**Background:**

We explored the heterogeneity of philadelphia chromosome-positive acute lymphoblastic leukemia (Ph1-ALL) in a study of the effect of early features on prognosis in children. Here we report the long-term results of the FRALLE 93 study conducted in the era before the use of tyrosine kinase inhibitors.

**Methods:**

Between 1993 and 1999, 36 children with Ph1-ALL were enrolled into the FRALLE 93 protocol. After conventional four-drug induction, children were stratified by availability of an HLA-matched sibling.

**Results:**

Complete remission (CR) was observed in 26 children (72%), of which 13 underwent allogeneic bone marrow transplantation (BMT). Thirty-one children were good responders to prednisone, defined on day 8, and 21 were good responders to chemotherapy, defined by day-21 bone marrow (M1). Overall five-year disease-free survival (DFS) was 42 ± 9.7%. Based on multivariate analysis, two groups showed marked differences in five-year outcome: children with age<10, leukocyte count <100,000/mm^3 ^and day-21 M1 marrow had a more favorable prognosis (14 pts: 100% CR, event free survival [EFS]: 57%, overall survival [OS]: 79%), than the high-risk group (22 patients: 55% CR, EFS: 18%, OS: 27%) (p < 0.005). We also observed a non statistically significant difference (p = 0.14) in outcome between these groups for transplanted patients (5-year DFS: 83 ± 14% and 33 ± 15%, respectively).

**Conclusion:**

Age, leukocyte count and early response to treatment defined by the D21 bone marrow response provide an accurate model for outcome prediction. The combination of available tools such as minimal residual disease assessment with determination of these simple factors could be useful for refining indications for BMT in the current era of tyrosine-kinase inhibitor-based therapy.

## Background

The philadelphia chromosome (Ph1) is detectable in 2% to 5% of children with acute lymphoblastic leukemia (ALL) [1,2]. The detection of a philadelphia chromosome remains a major prognostic factor of induction failure. Despite the steady improvement in the management of ALL in children, Ph1-ALL is associated with high rates of relapse or resistance to treatment [3-5]. This disease is heterogeneous in terms of clinical parameters such as leukocyte count, age at diagnosis, and initial steroid response [4,6]. A slow early response to conventional therapy has also been reported as indicative of a poor prognosis [2].

Recent gene expression studies have identified a heterogeneous pattern of expression associated with *BCR-ABL *status, which may be useful for developing novel prognostic markers and future patient stratification procedures [7-9]. New therapeutic agents such as tyrosine kinase inhibitors (imatinib and dasatinib) have been developed and yield good results in adults with Ph1-ALL [10-12]. Little information on the use of these drugs in children has been reported; the identification of predictors of responsiveness to early conventional treatment may thus be beneficial for the accurate stratification of children and for improving outcome [13,14].

We studied the impact of the National Cancer Institute (NCI) risk factors and steroid and early chemotherapy responses in 36 children with untreated Ph1-ALL enrolled in the FRALLE 93 trial between 1993 and 1999.

## Methods

The FRALLE 93 trial was open to children aged 0 to 20 years with untreated ALL, not including those with L3 ALL or Down's syndrome. Between June 1, 1993, and December 31, 1999, 1395 children were enrolled onto the FRALLE 93 trial in 18 French pediatric centers and one Belgian pediatric center. This study was approved by the ethics committee of the hôpital Saint Louis, France (accepted April 29, 1993). All patients, or their parents, provided informed consent in accordance with the Declaration of Helsinki. The diagnosis of ALL was based on morphological, immunophenotypic and cytogenetic analyses of bone marrow samples. From 1994, children were systematically screened for four fusion transcripts (*TEL-AML1, BCR-ABL, E2A-PBX1, MLL-AF4*).

### Stratification and treatment

Patients carrying t(9,22) or *BCR-ABL *were assigned to the very high risk group of the FRALLE 93 trial (Table 1) [5]. Patients received initial treatment comprising a prednisone prophase and a triple-drug intrathecal injection. Induction treatment then included prednisone, vincristine, L-asparaginase, a 120 mg/m2 cumulative dose of daunorubicin (increased to 160 mg/m2 after July 1996) and two more triple-drug intrathecal injections. Treatment was then stratified according to availability of an HLA-matched sibling. Children with an HLA-matched sibling received alternating courses of R3 (Cytarabine, Etoposide, Dexamethasone) and COPADM (Vincristine, Methotrexate, Doxorubicin, Cyclophosphamide, Prednisone) therapy (for a total of 3 courses of treatment) before an allogeneic bone marrow transplantation (Table 1). The remaining children with no sibling donors were eligible for either autologous transplantation after six courses of treatment (with graft harvesting carried out after the fifth course of chemotherapy) or non genoidentical allogeneic transplantation.

**Table 1:** FRALLE 93 protocol schedule for very high risk patients

Phase	Treatment	Dose
Induction	Vincristine	1.5 mg/m^2 ^IV (max, 2 mg) on D8, D15, D22, D29
	Prednisone	60 mg/m^2^/d PO on D1–8, 40 mg/m^2^/d on D8–28
	Daunorubicin^§^	40 mg/m^2 ^IV on D8, D9, D10, D15
	L-Asparaginase^§^	10,000 U/m^2 ^IM or IV on D20, D22, D24, D26, D29, D31, D33, D35
	intrathecal therapy*	before D4, D8, and D15
R3 course	Cytarabine	2 g/m^2 ^IV bid on D1–D2
	Etoposide	150 mg/m^2 ^IV on D3, D4, D5
	Dexamethasone	20 mg/m^2 ^PO on D2–D5
	intrathecal therapy*	D5
COPADM course	Vincristine	1.5 mg/m^2 ^(max, 2 mg) IV on D1
	Methotrexate	8000 mg/m^2 ^(24-hour IV infusion with leucovorin rescue) on D1
	Doxorubicin	60 mg/m^2 ^IV on D2
	Cyclophosphamide	375 mg/m^2 ^bid on D2–D3
	Prednisone	60 mg/m^2 ^PO on D1–D5
Stem-cell transplantation	age >4 years: TBI	2 Gy bid on D-9 to D-7
	age <4 years: Busulfan	30 mg/m^2^/6 hours PO on D-10 to D-7
	All patients: Cytarabine	3 g/m^2 ^bid on D-5 to D-4
	Melphalan	140 mg/m^2^IV on D-2
	Transplantation	IV day 0
Maintenance after autologous transplantation	6-mercaptopurine Vincristine	75 mg/m^2^/d 2 years after complete remission 1.5 mg/m^2 ^monthly for 12 months

Abbreviations: PO, per os; IV, intravenous; IM, intramuscular; max, maximum; TBI, total body irradiation

^§^before July 1996: daunorubicin on D8, D15, and D22 and L-Asparaginase on D22, D24, D26, D29, D31, D33.

*Methotrexate, Cytarabine, and Depomedrol in doses based on patient age

Other post-induction regimen: no R3 or COPADM but 1 consolidation, 2 intensifications, and an interim phase over 36 weeks before 18 months of maintenance therapy

Treatment outcome was analyzed according to indicators of early response to therapy. The prognostic value of persistent lymphoblasts in blood sampled at day 8 and in bone marrow at day 21–22 has been demonstrated in several previous studies [15-17]. We also recently showed that the persistence of lymphoblasts in bone marrow on day 21 of four-drug induction was associated with a higher risk of induction failure [5]. A good prednisone response was defined as a blast count <1000/μl blood after the first seven days on prednisone therapy (i.e. on day 8) and one triple-drug intrathecal injection. A poor prednisone response was defined by a blast count ≥ 1000/μl on day 8. A good early response to chemotherapy was defined by a blast count fewer than 5% in bone marrow smears on day 21 (M1) and a poor early chemotherapy response by a blast count equal to or more than 5% (with two categories: M2 = 5% to 25% and M3 = more than 25%). Complete remission (CR) was defined by no physical evidence of disease, no detectable leukemic blasts on blood smears and less than 5% blasts on bone marrow smears, active hematopoiesis and normal cerebrospinal fluid.

### Statistical analysis

Analysis was based on an intent-to-treat principle. CR rates were compared using Fisher's exact test. Censored endpoints were estimated by the non parametric Kaplan-Meier method, and then compared by the log-rank test. Multivariate analyses were carried out to define the set of informative prognostic factors, using regression models adapted to the endpoint, namely the logistic model for CR rates, and Cox model for overall survival (OS) and event free survival (EFS).

Type I error was fixed at 5%. All tests were two-tailed. Statistical analysis was performed using SAS 9.1 (SAS, Inc, Cary, NC).

## Results

Ph1 was detected by conventional cytogenetic analysis (t(9;22) (q34;q11)) or a *BCR-ABL *rearrangement was detected by reverse transcriptase-polymerase chain reaction (RT-PCR) in 3% of the B-lineage ALL. Of the 36 Ph1-ALL children, 30 had detectable t(9;22) and six were identified solely by the presence of the *BCR-ABL *fusion transcript. Thus, fusion transcript analysis was able to detect nearly 17% more Ph1 patients than conventional karyotype. The m-*BCR *breakpoint was detected in 30 patients and the M-*BCR *breakpoint in three others (3 patients were identified solely by the detection of t(9;22)). At baseline, median age was eight years [range: 0.70–19.5; Q1–Q3: 4.35 to 12.35], male-to-female ratio was 1.1, median leukocyte count was 29.1 × 10^9^/l [Q1–Q3: 12.5–137] and median hemoglobin was 9 g/dl [2.5–15]; four patients displayed central nervous system defects (11.1%).

Complete remission was observed in 26 children (72%), after a median of 40 days [range 32–51] consistent with previous findings [2,4]. We did not find any differences between the effects of including patients at two different time periods (before or after July 1996) and the addition of one dose of daunorubicin and two doses of asparaginase (but the numbers of children within each group are small) (Table 2).

**Table 2:** Outcome of the 36 children with philadelphia chromosome-positive acute lymphoblastic leukemia as a function of early characteristics

	No patients	No CR (%)	*p*	5 yr-EFS	*p*	5 yr-OS	*p*
**Whole cohort**	N = 36	26 (72%)		33.3 ± 7.9		47.2 ± 8.3	

**Inclusion period**							
< July, 1996	15	9 (60%)		20 ± 10		33 ± 12	
> July, 1996	21	17 (81%)	0.26	43 ± 11	0.13	57 ± 11	0.31
**Age (years)**							
<10	23	20 (87%)		48 ± 10		61 ± 10	
≥ 10	13	6 (46%)	0.018	15 ± 10	0.01	23 ± 12	0.006
**WBC (/mm**^3^**)**							
<50,000	21	17 (81%)		33 ± 10			
≥ 50,000	15	9 (60%)	0.26	33 ± 12	0.54		0.91
<100,000	26	22 (85%)		42 ± 10		58 ± 10	
≥ 100,000	10	4 (40%)	0.014	10 ± 9	0.003	20 ± 13	0.03
**Response to steroid (+3 drug intra-thecal injection) at D8**							
poor (≥ 1000 blasts/mm^3^)	5	2 (40%)		20 ± 18		20 ± 18	
good(<1000 blasts/mm^3^)	31	24 (77%)	0.12	35 ± 9	0.33	52 ± 9	0.19
**Response to chemotherapy evaluated on D21**							
M1(≤ 5% blasts)	21	20 (95%)		43 ± 11		62 ± 11	
M2+M3 (>5%–25% and >25% blasts)	15	6 (40%)	0.0004	20 ± 10	0.18	27 ± 11	0.06
**Age, D21 response and WBC**							
Age<10 and D21 M1 and WBC count<100,000	14	14 (100%)		57 ± 13		79 ± 11	
Others	22	12 (55%)	0.003	18 ± 8	0.002	27 ± 9	0.003

WBC = white blood cell; D = day; Response to steroid is evaluated in peripheral blood on day 8; Response to chemotherapy is evaluated in bone marrow on day 21

**Figure 1:**
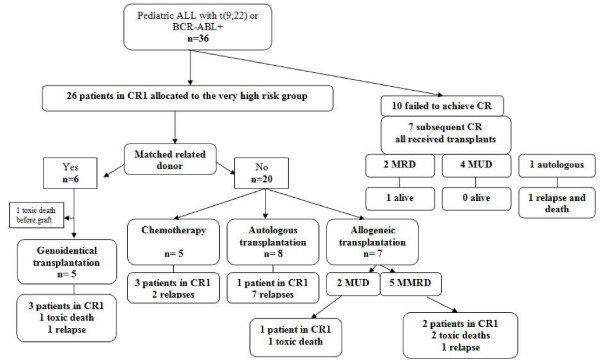
**flowchart of the FRALLE 93 trial for philadelphia chromosome-positive ALL**. Children with ALL were allocated to very high risk group as soon as a t(9;22) or *BCR-ABL *rearrangement was detected. Post-induction treatment was then stratified according to availability of an HLA-matched sibling. CR1 = first complete remission; MRD = matched related donor; MUD = matched unrelated donor; MMRD = mismatched related donor.

Ten patients did not achieve CR after induction therapy, but CR was observed in seven of them after one further course of chemotherapy. The dexamethasone, cytarabine, cyclophosphamide, etoposide, and idarubicin or daunorubicin (CAZED) scheme was recommended as salvage treatment in the FRALLE 93 protocol and thus was used in five of these seven patients (amsacrine and cytarabine for the 2 others) [5]. All seven children were then transplanted (2 matched related transplants, 4 matched unrelated transplants and one autologous transplantation); outcomes are described in Figure 1. Of the other children with CR observed after induction, five of the six children with an HLA-matched sibling (MRD) received alternate courses of R3 and COPADM treatment before allogeneic bone marrow transplantation (one toxic death occurred before graft). Eight children received an autologous transplant, five a mismatched related transplant and two others a matched unrelated transplant (MUD) (Figure 1). Five other CR patients received a different post-induction regimen with intensified chemotherapy, including one consolidation, a double delayed intensification, and an interim phase over 36 weeks before 18 months of maintenance therapy.

Patient follow-up data were updated in February, 2007. In an intention-to-treat analysis, 10 children remained in CR1, giving a five-year disease-free survival (DFS) rate of 42.3% ± 9.7% and a five-year OS rate of 47.2% ± 8.3. Leukemic relapse was the most common cause of adverse events. Eleven children relapsed (no late relapse after 5 years) and five toxic deaths occurred (including one late death from infection). No secondary malignancy was observed.

**Figure 2:**
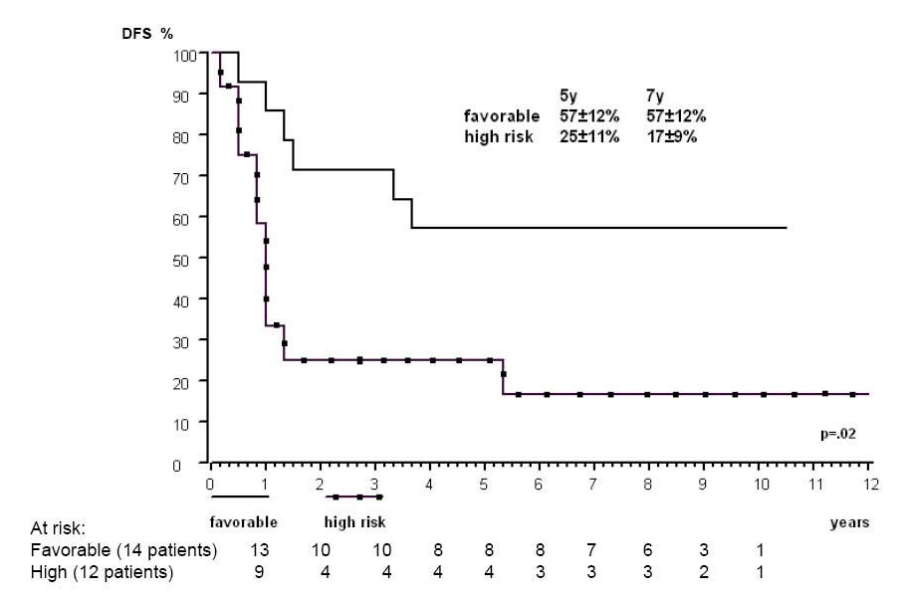
**Kaplan-Meier five- and seven-year DFS analysis based on risk group (n = 26)**. The multivariable regression model revealed the independent prognostic value of age<10 years, M1 bone marrow and WBC count<100,000/mm^3^, defining the group with favorable outcome.

Table 2 summarizes the main endpoints – CR, EFS and survival – overall and as a function of the main patient characteristics. Based on univariate analyses using a 5% significance level for all endpoints, we identified two predictors of poor outcome: age ≥ 10 and white blood cells (WBC) count >100,000/mm^3^. Response to chemotherapy based on day 21 bone marrow was only predictive of induction response (p = 0.0004). When incorporated simultaneously in multivariate regression models, only one predictive factor retained significant prognostic value for a particular endpoint: M1 for CR, WBC count >100,000/mm^3 ^for EFS and age ≥ 10 for OS. Combining the information of these three binary variables defined two groups differing widely in terms of outcome: the 14 children with age<10, WBC<100,000/mm^3 ^and M1 defined a group with a favorable prognosis (100% CR, 5-yr EFS: 57%, 5-yr OS: 79%), whereas the remaining 22 children had poorer outcomes (55% CR, 5-yr EFS: 18%, 5-yr OS: 27%) (p = 0.003, 0.002, and 0.003, respectively) (Figure 2). These prognostic factors remain important in the transplanted patients (equally divided in both groups), even if significance is not reached due to the small number of patients (5-yr DFS: 83 ± 14% for patients with age<10, WBC<100,000/mm^3 ^and M1 and 33 ± 15% for the remaining patients) (p = 0.14).

## Discussion and conclusion

This long-term study, carried out before the introduction of imatinib mesylate, confirmed the prognostic value of two major clinical factors (age, WBC count at diagnosis) and identified the D21 marrow response as a powerful complementary tool. We did not find prednisone response to have statistically significant predictive value for any endpoint studied, in contrast with earlier reports [4,6]. This could be explained by the small sample size, and the small number of poor prednisone responders (5 of 36; 14%). However, in our protocol the combination of a three-drug intrathecal injection (methotrexate, cytarabin and depomedrol) on day 1 with the 7 day prednisone prophase prevents the comparison of our steroid-resistance data to data in other studies using intrathecal methotrexate.

Based on these three easily available indicators (age, WBC at diagnosis and D21 response to chemotherapy), a predictive model can be built to define two subsets of children that differ widely in terms of outcome. Such a model should be further investigated in larger samples and in ongoing pediatric trials integrating tyrosine-kinase inhibitors. The improved early responses observed with imatinib or dasatinib in adult studies, and similar, but very preliminarily, results obtained in one pediatric study with imatinib, now question the appropriate use of allogeneic stem-cell transplantation [14,18]. Indeed, the COG AALL0031 study showed that continuous administration of imatinib given in combination with intensive chemotherapy backbone resulted in a significant improvement in early EFS. More specifically cohort 5, who received 340 mg/m2 of imatinib for 280 days with chemotherapy only, had a similar two years EFS as compared to the cohort of patients who underwent stem cell transplantation (32 patients) either according to the protocol (MRD-21 patients) or from MUD (11 patients)[14]. The combination of available tools, including minimal residual disease assessment, with these easily measured predictive features could be useful for refining the indications for bone marrow transplantation [19-22]. Thus, if the long term follow-up of the AALL0031 non randomized study was confirmed, good risk patients could be spared by transplantation.

## Competing interests

The authors declare that they have no competing interests.

## Authors' contributions

All the authors have substantially contributed to the conception and design or acquisition of data or analysis and interpretation of the data in this multicentric study, and participated to drafting and revising the article. SC and MFA performed statistical analysis. All authors read and approved the final manuscript.

## Pre-publication history

The pre-publication history for this paper can be accessed here:

http://www.biomedcentral.com/1471-2407/9/14/prepub
